# *catena*-Poly[[trimethyl­tin(IV)]-μ-2-(2-chloro­phenyl)­acetato]

**DOI:** 10.1107/S1600536809038872

**Published:** 2009-09-30

**Authors:** Liyuan Wen, Handong Yin, Wenkuan Li

**Affiliations:** a College of Chemistry and Chemical Engineering, Liaocheng University, Shandong 252059, People’s Republic of China

## Abstract

In the title polymeric coordination compound, [Sn(CH_3_)_3_(C_8_H_6_ClO_2_)]_*n*_, the Sn atoms exhibit a distorted trigonal-bipyramidal geometry with the carboxyl­ate O atoms of the 2-chloro­phenyl­acetato ligands in axial positions and with the equatorial sites occupied by the three methyl groups. Adjacent Sn atoms are bridged by coordination to the two O atoms of each 2-chloro­phenyl­acetato ligand, forming a chain structure.

## Related literature

For the biological activity of organotin compounds, see: Wang *et al.* (2007 ▶). For related structures, see: Wang *et al.* (2007 ▶); Ma *et al.* (2006 ▶).
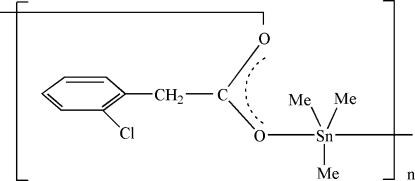


## Experimental

### 

#### Crystal data

[Sn(CH_3_)_3_(C_8_H_6_ClO_2_)]
*M* *_r_* = 333.39
Monoclinic, 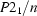

*a* = 7.0754 (9) Å
*b* = 28.306 (3) Å
*c* = 13.6721 (15) Å
β = 93.117 (2)°
*V* = 2734.1 (5) Å^3^
*Z* = 8
Mo *K*α radiation
μ = 2.05 mm^−1^
*T* = 298 K
0.49 × 0.32 × 0.15 mm

#### Data collection

Siemens SMART CCD area-detector diffractometer
Absorption correction: multi-scan (*SADABS*; Sheldrick, 1996 ▶) *T*_min_ = 0.434, *T*_max_ = 0.749
14063 measured reflections
4820 independent reflections
3411 reflections with *I* > 2σ(*I*)
*R*_int_ = 0.056

#### Refinement

*R*[*F* ^2^ > 2σ(*F* ^2^)] = 0.047
*wR*(*F* ^2^) = 0.107
*S* = 1.05
4820 reflections
277 parameters
H-atom parameters constrained
Δρ_max_ = 1.09 e Å^−3^
Δρ_min_ = −0.92 e Å^−3^

### 

Data collection: *SMART* (Siemens, 1996 ▶); cell refinement: *SAINT* (Siemens, 1996 ▶); data reduction: *SAINT*; program(s) used to solve structure: *SHELXS97* (Sheldrick, 2008 ▶); program(s) used to refine structure: *SHELXL97* (Sheldrick, 2008 ▶); molecular graphics: *SHELXTL* (Sheldrick, 2008 ▶); software used to prepare material for publication: *SHELXTL*.

## Supplementary Material

Crystal structure: contains datablocks I, global. DOI: 10.1107/S1600536809038872/sj2651sup1.cif

Structure factors: contains datablocks I. DOI: 10.1107/S1600536809038872/sj2651Isup2.hkl

Additional supplementary materials:  crystallographic information; 3D view; checkCIF report

## Figures and Tables

**Table 1:** Selected bond lengths (Å)

Sn1—C17	2.107 (7)
Sn1—C18	2.117 (7)
Sn1—C19	2.120 (7)
Sn1—O3	2.194 (4)
Sn1—O1	2.396 (5)
Sn2—C22	2.102 (7)
Sn2—C20	2.113 (7)
Sn2—C21	2.117 (7)
Sn2—O2	2.207 (5)
Sn2—O4^i^	2.432 (5)

Symmetry code: (i) 
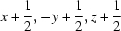
.

**Fig. 1.**
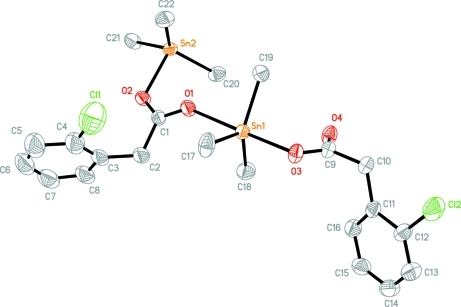
The asymmetric unit of the title compound, showing 50% probability displacement ellipsoids. H atoms have been omitted for clarity.

**Fig. 2.**
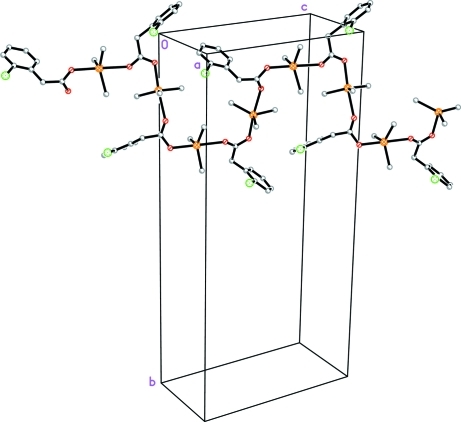
A view of the one-dimensional extended chain structure in the title compound.

## supplementary crystallographic information

### Comment

The chemistry of organotin(IV) derivatives is a subject of study with growing interest due to their significant antimicrobial properties as well as antitumor activities (Wang *et al.*, 2007). As a part of our ongoing investigations in this field we have synthesized the title compound and present its crystal structure here. The title compound, which is shown in Fig.1 forms an extended one-dimensional chain structure arising from Sn—O bridges formed by the 2-(2-chlorophenyl)acetato ligands. The Sn—O bond distances in the compound (Sn(1)—O(1) = 2.396 (5) Å; Sn(1)—O(3) = 2.194 (4) Å) are comparable to those found in related organotin carboxylates (Ma *et al.*, 2006). The Sn atom assumes a slightly distorted trigonal-bipyramidal coordination geometry, provided by and three methyl groups in the equatorial positions and two O atoms of symmetry related carboxylate groups in the axial positions.

### Experimental

The reaction was carried out under a nitrogen atmosphere. 2-(2-chlorophenyl)acetic acid (1 mmol) and sodium ethoxide (1.2 mmol) were added to a stirred solution of benzene (30 ml) in a Schlenk flask and stirred for 0.5 h. Trimethyltin chloride (1 mmol) was then added to the reactor and the reaction mixture was stirred for 12 h at room temperature. The resulting clear solution was evaporated under vacuum. The product was crystallized from a solution of dichloromethane/methanol (1:1) to yield colourless blocks of the title compound (yield 81%. m.p.390 K). Anal. Calcd (%) for C_11_H_15_Cl_1_O_2_Sn_1_ (Mr = 333.37): C,39.63; H, 4.54; Cl, 10.63. Found (%): C, 39.51; H, 4.64; Cl, 10.75.

### Refinement

The H atoms were positioned geometrically, with methyl C—H distances of 0.96 Å and aromatic C—H distances of 0.93 Å, and refined as riding on their parent atoms, with *U*_iso_(H) = 1.2 *U*_eq_(C, O) or 1.5 *U*_eq_(C) for the methyl groups.

### Crystal data

**Table:**

[Sn(CH_3_)_3_(C_8_H_6_ClO_2_)]	*F*(000) = 1312
*M**_r_* = 333.39	*D*_x_ = 1.620 Mg m^−^^3^
Monoclinic, *P*2_1_/*n*	Mo *K*α radiation, λ = 0.71073 Å
Hall symbol: -P 2yn	Cell parameters from 4326 reflections
*a* = 7.0754 (9) Å	θ = 2.6–24.8°
*b* = 28.306 (3) Å	µ = 2.05 mm^−^^1^
*c* = 13.6721 (15) Å	*T* = 298 K
β = 93.117 (2)°	Block, colourless
*V* = 2734.1 (5) Å^3^	0.49 × 0.32 × 0.15 mm
*Z* = 8	

### Data collection

**Table:**

Siemens SMART CCD area-detector diffractometer	4820 independent reflections
Radiation source: fine-focus sealed tube	3411 reflections with *I* > 2σ(*I*)
graphite	*R*_int_ = 0.056
φ and ω scans	θ_max_ = 25.0°, θ_min_ = 1.7°
Absorption correction: multi-scan (*SADABS*; Sheldrick, 1996)	*h* = −8→8
*T*_min_ = 0.434, *T*_max_ = 0.749	*k* = −33→33
14063 measured reflections	*l* = −10→16

### Refinement

**Table:**

Refinement on *F*^2^	Primary atom site location: structure-invariant direct methods
Least-squares matrix: full	Secondary atom site location: difference Fourier map
*R*[*F*^2^ > 2σ(*F*^2^)] = 0.047	Hydrogen site location: inferred from neighbouring sites
*wR*(*F*^2^) = 0.107	H-atom parameters constrained
*S* = 1.05	*w* = 1/[σ^2^(*F*_o_^2^) + (0.0224*P*)^2^ + 9.0463*P*] where *P* = (*F*_o_^2^ + 2*F*_c_^2^)/3
4820 reflections	(Δ/σ)_max_ = 0.001
277 parameters	Δρ_max_ = 1.09 e Å^−^^3^
0 restraints	Δρ_min_ = −0.92 e Å^−^^3^

### Fractional atomic coordinates and isotropic or equivalent isotropic displacement parameters (Å^2^)

**Table:**

	*x*	*y*	*z*	*U*_iso_*/*U*_eq_	
Sn1	0.02624 (7)	0.138217 (16)	0.88059 (3)	0.04764 (16)	
Sn2	0.20583 (7)	0.212967 (17)	1.21572 (3)	0.04848 (16)	
Cl1	0.4073 (6)	0.03321 (16)	1.1310 (3)	0.1655 (15)	
Cl2	−0.2267 (4)	0.14040 (10)	0.36127 (15)	0.0943 (8)	
O1	0.1070 (7)	0.14009 (16)	1.0530 (3)	0.0583 (13)	
O2	0.1197 (7)	0.13802 (16)	1.2145 (3)	0.0572 (13)	
O3	−0.0431 (7)	0.13733 (16)	0.7223 (3)	0.0564 (13)	
O4	−0.1907 (7)	0.20535 (18)	0.7318 (3)	0.0620 (14)	
C1	0.0842 (11)	0.1192 (3)	1.1319 (5)	0.0553 (19)	
C2	0.0044 (13)	0.0697 (3)	1.1322 (6)	0.073 (2)	
H2A	−0.1323	0.0720	1.1333	0.088*	
H2B	0.0328	0.0546	1.0710	0.088*	
C3	0.0721 (17)	0.0382 (3)	1.2138 (7)	0.079 (3)	
C4	0.2486 (19)	0.0191 (4)	1.2200 (8)	0.103 (3)	
C5	0.316 (2)	−0.0112 (4)	1.2941 (10)	0.127 (4)	
H5	0.4382	−0.0237	1.2975	0.153*	
C6	0.184 (3)	−0.0208 (4)	1.3620 (10)	0.121 (5)	
H6	0.2224	−0.0410	1.4131	0.145*	
C7	0.001 (2)	−0.0036 (4)	1.3623 (9)	0.115 (4)	
H7	−0.0816	−0.0121	1.4100	0.138*	
C8	−0.0504 (18)	0.0264 (3)	1.2880 (8)	0.100 (3)	
H8	−0.1708	0.0397	1.2861	0.120*	
C9	−0.1339 (10)	0.1720 (3)	0.6832 (5)	0.0504 (18)	
C10	−0.1744 (10)	0.1690 (3)	0.5734 (5)	0.0553 (19)	
H10A	−0.0690	0.1534	0.5443	0.066*	
H10B	−0.1837	0.2007	0.5469	0.066*	
C11	−0.3557 (10)	0.1423 (2)	0.5447 (5)	0.0514 (18)	
C12	−0.3923 (11)	0.1278 (3)	0.4478 (6)	0.062 (2)	
C13	−0.5520 (14)	0.1049 (3)	0.4182 (7)	0.079 (3)	
H13	−0.5713	0.0958	0.3531	0.095*	
C14	−0.6863 (13)	0.0950 (3)	0.4838 (7)	0.080 (3)	
H14	−0.7967	0.0792	0.4633	0.097*	
C15	−0.6576 (12)	0.1084 (3)	0.5797 (7)	0.072 (2)	
H15	−0.7488	0.1020	0.6244	0.086*	
C16	−0.4919 (11)	0.1317 (3)	0.6100 (6)	0.064 (2)	
H16	−0.4723	0.1403	0.6754	0.077*	
C17	0.1855 (13)	0.0758 (3)	0.8683 (6)	0.079 (3)	
H17A	0.2891	0.0759	0.9167	0.119*	
H17B	0.2338	0.0741	0.8041	0.119*	
H17C	0.1062	0.0489	0.8785	0.119*	
C18	−0.2588 (11)	0.1335 (3)	0.9193 (6)	0.077 (2)	
H18A	−0.3086	0.1030	0.9012	0.115*	
H18B	−0.3322	0.1576	0.8855	0.115*	
H18C	−0.2649	0.1378	0.9887	0.115*	
C19	0.1885 (11)	0.2012 (3)	0.8801 (5)	0.069 (2)	
H19A	0.3035	0.1969	0.9196	0.103*	
H19B	0.1171	0.2266	0.9062	0.103*	
H19C	0.2183	0.2086	0.8141	0.103*	
C20	−0.0375 (10)	0.2385 (3)	1.1363 (6)	0.067 (2)	
H20A	−0.0001	0.2560	1.0803	0.100*	
H20B	−0.1072	0.2588	1.1776	0.100*	
H20C	−0.1158	0.2124	1.1149	0.100*	
C21	0.2034 (13)	0.2164 (3)	1.3703 (5)	0.075 (3)	
H21A	0.1478	0.2458	1.3891	0.112*	
H21B	0.3308	0.2145	1.3980	0.112*	
H21C	0.1306	0.1906	1.3939	0.112*	
C22	0.4641 (10)	0.2008 (3)	1.1510 (6)	0.071 (2)	
H22A	0.4750	0.1678	1.1356	0.107*	
H22B	0.5669	0.2099	1.1957	0.107*	
H22C	0.4684	0.2191	1.0920	0.107*	

### Atomic displacement parameters (Å^2^)

**Table:**

	*U*^11^	*U*^22^	*U*^33^	*U*^12^	*U*^13^	*U*^23^
Sn1	0.0547 (3)	0.0418 (3)	0.0458 (3)	0.0031 (2)	−0.0036 (2)	−0.0021 (2)
Sn2	0.0490 (3)	0.0512 (3)	0.0451 (3)	−0.0101 (2)	0.0020 (2)	−0.0036 (2)
Cl1	0.138 (3)	0.195 (4)	0.163 (3)	0.045 (3)	0.005 (3)	0.002 (3)
Cl2	0.1043 (18)	0.130 (2)	0.0492 (12)	−0.0172 (16)	0.0086 (12)	−0.0131 (13)
O1	0.083 (4)	0.046 (3)	0.045 (3)	−0.007 (3)	−0.002 (2)	0.000 (2)
O2	0.083 (4)	0.047 (3)	0.041 (3)	−0.015 (3)	−0.001 (2)	−0.003 (2)
O3	0.074 (3)	0.049 (3)	0.045 (3)	0.017 (3)	−0.008 (2)	−0.007 (2)
O4	0.073 (4)	0.058 (3)	0.054 (3)	0.022 (3)	−0.009 (3)	−0.005 (3)
C1	0.071 (5)	0.046 (4)	0.048 (4)	−0.003 (4)	−0.007 (4)	0.001 (4)
C2	0.102 (7)	0.057 (5)	0.059 (5)	−0.019 (5)	−0.013 (5)	0.001 (4)
C3	0.122 (9)	0.044 (5)	0.068 (6)	−0.019 (5)	−0.016 (6)	−0.006 (4)
C4	0.145 (11)	0.071 (7)	0.088 (7)	−0.001 (7)	−0.021 (8)	−0.002 (6)
C5	0.181 (14)	0.084 (9)	0.113 (10)	0.012 (9)	−0.035 (10)	−0.006 (8)
C6	0.195 (16)	0.064 (8)	0.101 (10)	−0.014 (9)	−0.027 (10)	0.006 (7)
C7	0.178 (14)	0.078 (8)	0.089 (8)	−0.040 (9)	−0.007 (9)	−0.002 (7)
C8	0.153 (10)	0.060 (6)	0.084 (7)	−0.026 (6)	−0.011 (7)	−0.003 (6)
C9	0.051 (4)	0.057 (5)	0.043 (4)	0.007 (4)	−0.002 (3)	0.001 (4)
C10	0.055 (5)	0.063 (5)	0.047 (4)	0.003 (4)	−0.006 (3)	−0.001 (4)
C11	0.050 (4)	0.053 (5)	0.051 (4)	0.007 (3)	−0.007 (3)	0.000 (3)
C12	0.064 (5)	0.061 (5)	0.060 (5)	0.002 (4)	−0.009 (4)	−0.010 (4)
C13	0.087 (7)	0.074 (6)	0.074 (6)	−0.010 (5)	−0.012 (5)	−0.010 (5)
C14	0.077 (6)	0.068 (6)	0.094 (7)	−0.012 (5)	−0.012 (5)	−0.002 (5)
C15	0.063 (5)	0.070 (6)	0.083 (6)	−0.010 (4)	0.008 (5)	0.005 (5)
C16	0.068 (5)	0.066 (5)	0.058 (5)	0.000 (4)	0.000 (4)	0.000 (4)
C17	0.106 (7)	0.071 (6)	0.060 (5)	0.036 (5)	−0.013 (5)	−0.009 (4)
C18	0.059 (5)	0.093 (7)	0.078 (6)	−0.008 (5)	0.006 (4)	0.006 (5)
C19	0.074 (5)	0.075 (6)	0.057 (5)	−0.017 (4)	0.000 (4)	0.012 (4)
C20	0.050 (5)	0.063 (5)	0.086 (6)	0.003 (4)	0.001 (4)	−0.010 (4)
C21	0.117 (7)	0.068 (6)	0.039 (4)	−0.029 (5)	0.005 (4)	−0.004 (4)
C22	0.057 (5)	0.082 (6)	0.076 (5)	−0.006 (4)	0.006 (4)	−0.003 (5)

### Geometric parameters (Å, °)

**Table:**

Sn1—C17	2.107 (7)	C10—C11	1.522 (9)
Sn1—C18	2.117 (7)	C10—H10A	0.9700
Sn1—C19	2.120 (7)	C10—H10B	0.9700
Sn1—O3	2.194 (4)	C11—C16	1.381 (10)
Sn1—O1	2.396 (5)	C11—C12	1.397 (10)
Sn2—C22	2.102 (7)	C12—C13	1.345 (11)
Sn2—C20	2.113 (7)	C13—C14	1.371 (12)
Sn2—C21	2.117 (7)	C13—H13	0.9300
Sn2—O2	2.207 (5)	C14—C15	1.369 (11)
Sn2—O4^i^	2.432 (5)	C14—H14	0.9300
Cl1—C4	1.746 (12)	C15—C16	1.389 (11)
Cl2—C12	1.747 (8)	C15—H15	0.9300
O1—C1	1.249 (8)	C16—H16	0.9300
O2—C1	1.263 (8)	C17—H17A	0.9600
O3—C9	1.273 (8)	C17—H17B	0.9600
O4—C9	1.235 (8)	C17—H17C	0.9600
O4—Sn2^ii^	2.432 (5)	C18—H18A	0.9600
C1—C2	1.510 (10)	C18—H18B	0.9600
C2—C3	1.487 (11)	C18—H18C	0.9600
C2—H2A	0.9700	C19—H19A	0.9600
C2—H2B	0.9700	C19—H19B	0.9600
C3—C4	1.359 (14)	C19—H19C	0.9600
C3—C8	1.411 (13)	C20—H20A	0.9600
C4—C5	1.395 (15)	C20—H20B	0.9600
C5—C6	1.380 (17)	C20—H20C	0.9600
C5—H5	0.9300	C21—H21A	0.9600
C6—C7	1.387 (17)	C21—H21B	0.9600
C6—H6	0.9300	C21—H21C	0.9600
C7—C8	1.358 (14)	C22—H22A	0.9600
C7—H7	0.9300	C22—H22B	0.9600
C8—H8	0.9300	C22—H22C	0.9600
C9—C10	1.515 (9)		
			
C17—Sn1—C18	119.2 (4)	C9—C10—H10B	108.9
C17—Sn1—C19	114.4 (4)	C11—C10—H10B	108.9
C18—Sn1—C19	125.2 (3)	H10A—C10—H10B	107.7
C17—Sn1—O3	90.2 (2)	C16—C11—C12	116.7 (7)
C18—Sn1—O3	94.6 (3)	C16—C11—C10	123.3 (6)
C19—Sn1—O3	95.6 (2)	C12—C11—C10	120.0 (7)
C17—Sn1—O1	89.8 (2)	C13—C12—C11	122.4 (8)
C18—Sn1—O1	86.3 (3)	C13—C12—Cl2	118.5 (7)
C19—Sn1—O1	83.4 (2)	C11—C12—Cl2	119.1 (6)
O3—Sn1—O1	178.93 (18)	C12—C13—C14	120.1 (8)
C22—Sn2—C20	122.9 (3)	C12—C13—H13	119.9
C22—Sn2—C21	118.9 (3)	C14—C13—H13	119.9
C20—Sn2—C21	116.5 (3)	C15—C14—C13	119.9 (8)
C22—Sn2—O2	94.9 (3)	C15—C14—H14	120.1
C20—Sn2—O2	96.2 (3)	C13—C14—H14	120.1
C21—Sn2—O2	92.0 (2)	C14—C15—C16	119.7 (8)
C22—Sn2—O4^i^	86.0 (3)	C14—C15—H15	120.1
C20—Sn2—O4^i^	87.3 (2)	C16—C15—H15	120.1
C21—Sn2—O4^i^	83.4 (2)	C11—C16—C15	121.2 (7)
O2—Sn2—O4^i^	175.08 (16)	C11—C16—H16	119.4
C1—O1—Sn1	143.5 (5)	C15—C16—H16	119.4
C1—O2—Sn2	117.0 (4)	Sn1—C17—H17A	109.5
C9—O3—Sn1	119.0 (4)	Sn1—C17—H17B	109.5
C9—O4—Sn2^ii^	141.7 (5)	H17A—C17—H17B	109.5
O1—C1—O2	122.9 (7)	Sn1—C17—H17C	109.5
O1—C1—C2	120.6 (6)	H17A—C17—H17C	109.5
O2—C1—C2	116.5 (6)	H17B—C17—H17C	109.5
C3—C2—C1	116.9 (7)	Sn1—C18—H18A	109.5
C3—C2—H2A	108.1	Sn1—C18—H18B	109.5
C1—C2—H2A	108.1	H18A—C18—H18B	109.5
C3—C2—H2B	108.1	Sn1—C18—H18C	109.5
C1—C2—H2B	108.1	H18A—C18—H18C	109.5
H2A—C2—H2B	107.3	H18B—C18—H18C	109.5
C4—C3—C8	117.4 (10)	Sn1—C19—H19A	109.5
C4—C3—C2	122.9 (10)	Sn1—C19—H19B	109.5
C8—C3—C2	119.8 (10)	H19A—C19—H19B	109.5
C3—C4—C5	124.7 (13)	Sn1—C19—H19C	109.5
C3—C4—Cl1	119.3 (9)	H19A—C19—H19C	109.5
C5—C4—Cl1	116.0 (12)	H19B—C19—H19C	109.5
C6—C5—C4	113.0 (14)	Sn2—C20—H20A	109.5
C6—C5—H5	123.5	Sn2—C20—H20B	109.5
C4—C5—H5	123.5	H20A—C20—H20B	109.5
C5—C6—C7	127.0 (13)	Sn2—C20—H20C	109.5
C5—C6—H6	116.5	H20A—C20—H20C	109.5
C7—C6—H6	116.5	H20B—C20—H20C	109.5
C8—C7—C6	115.4 (13)	Sn2—C21—H21A	109.5
C8—C7—H7	122.3	Sn2—C21—H21B	109.5
C6—C7—H7	122.3	H21A—C21—H21B	109.5
C7—C8—C3	122.5 (12)	Sn2—C21—H21C	109.5
C7—C8—H8	118.8	H21A—C21—H21C	109.5
C3—C8—H8	118.8	H21B—C21—H21C	109.5
O4—C9—O3	122.4 (6)	Sn2—C22—H22A	109.5
O4—C9—C10	121.7 (6)	Sn2—C22—H22B	109.5
O3—C9—C10	115.9 (6)	H22A—C22—H22B	109.5
C9—C10—C11	113.2 (6)	Sn2—C22—H22C	109.5
C9—C10—H10A	108.9	H22A—C22—H22C	109.5
C11—C10—H10A	108.9	H22B—C22—H22C	109.5
			
C17—Sn1—O1—C1	−67.1 (9)	Cl1—C4—C5—C6	179.9 (9)
C18—Sn1—O1—C1	52.3 (9)	C4—C5—C6—C7	−0.1 (19)
C19—Sn1—O1—C1	178.4 (9)	C5—C6—C7—C8	−1.2 (19)
O3—Sn1—O1—C1	−159 (9)	C6—C7—C8—C3	2.0 (15)
C22—Sn2—O2—C1	−66.8 (6)	C4—C3—C8—C7	−1.6 (14)
C20—Sn2—O2—C1	57.1 (6)	C2—C3—C8—C7	177.3 (8)
C21—Sn2—O2—C1	174.0 (6)	Sn2^ii^—O4—C9—O3	160.1 (5)
O4^i^—Sn2—O2—C1	−167 (2)	Sn2^ii^—O4—C9—C10	−22.1 (12)
C17—Sn1—O3—C9	−174.5 (6)	Sn1—O3—C9—O4	−2.7 (9)
C18—Sn1—O3—C9	66.2 (6)	Sn1—O3—C9—C10	179.3 (4)
C19—Sn1—O3—C9	−60.0 (6)	O4—C9—C10—C11	−91.2 (9)
O1—Sn1—O3—C9	−83 (10)	O3—C9—C10—C11	86.7 (8)
Sn1—O1—C1—O2	−163.3 (5)	C9—C10—C11—C16	14.6 (10)
Sn1—O1—C1—C2	14.4 (13)	C9—C10—C11—C12	−166.5 (7)
Sn2—O2—C1—O1	4.5 (10)	C16—C11—C12—C13	0.3 (11)
Sn2—O2—C1—C2	−173.3 (5)	C10—C11—C12—C13	−178.7 (8)
O1—C1—C2—C3	148.0 (8)	C16—C11—C12—Cl2	179.6 (6)
O2—C1—C2—C3	−34.2 (11)	C10—C11—C12—Cl2	0.6 (10)
C1—C2—C3—C4	−72.9 (11)	C11—C12—C13—C14	0.1 (13)
C1—C2—C3—C8	108.2 (9)	Cl2—C12—C13—C14	−179.1 (7)
C8—C3—C4—C5	0.2 (15)	C12—C13—C14—C15	−0.1 (14)
C2—C3—C4—C5	−178.7 (9)	C13—C14—C15—C16	−0.4 (13)
C8—C3—C4—Cl1	−179.1 (7)	C12—C11—C16—C15	−0.8 (11)
C2—C3—C4—Cl1	2.0 (13)	C10—C11—C16—C15	178.1 (7)
C3—C4—C5—C6	0.6 (17)	C14—C15—C16—C11	0.9 (12)

Symmetry codes: (i) *x*+1/2, −*y*+1/2, *z*+1/2; (ii) *x*−1/2, −*y*+1/2, *z*−1/2.
